# The Diagnosis of Paternity

**Published:** 1912-12

**Authors:** R. H. L. Bibb


### EDITORIAL DEPARTMENT.

DR. F. E. DANIEL, Editor DR. R. H. L. BIBB, Associate Editor

DEPARTMENTS { EUGENICS: Drs. M. Duggan and T. Y. Hull.

WOMAN'S DEPARTMENT: Mrs. F. E. Daniel.

### The Diagnosis of Paternity.

The experiments of Mayoral and Jimenez (*La Médecine Scientifique*, February, 1912) do not assist very much in determining the paternity of Zebedee's children, nor do they lessen to any great extent the amount of wisdom required of a "son" to "know his own father."

Nevertheless, should the results of the experiments described below be confirmed by further experiments, and the test prove reliable, the discovery will have a tremendously important medico-legal bearing; for instance, in suits for divorce by the husband on the grounds of alleged infidelity, and in suits for seduction and breach of promise. It will also play an important part in determining whether or not a woman may carry a child more than nine months. In England, some years ago, a lord, an army officer, returned from India after eleven months' absence and found his lady with an infant of a few days old. He repudiated it, and a suit followed for divorce, the wife claiming that the baby was his, and the most noted specialists figured in the trial.

Basing their experiments upon the reaction of Elsberg in the early diagnosis of cancer, Mayoral and Jimenez have sought to apply it for diagnosis in the first months of pregnancy, and for determining the father in any given case of pregnancy.

Elsberg's cancer reaction, it will be remembered, is as follows: A cubic centimeter of red corpuscles is separated from the blood of a healthy individual, emulsionized with an isotonic solution of chloride of sodium and injected under the skin of the person suspected of cancer. If the subject thus injected be cancerous, an erythematous, somewhat painful, spot appears at the site of injection within six hours thereafter, provided the disease is not very far advanced.

Mayoral and Jimenez reason that pregnancy is analogous to cancer in that the pregnant contain large quantities of embryonic tissues with their respective antigens (receptors, heptophores, toxophores, zymophores, amboceptors, cytophiles and complements, almost *ad nauseam usque*), which, in response to the emulsionized red corpuscles, manifest their presence very much as does tuberculosis to Calmette's, Von Perquet's or Moro's tests for that disease. So they sought to ascertain:

1. If a pregnant woman, injected with an emulsion of the red corpuscles of the person who, supposedly, impregnated her, would give a positive Elsberg reaction.

2. If a pregnant female, injected with the corpuscles of a male who had never had sexual contact with her, would give the reaction.

3. If a woman, once fecundated by a certain man, would thereafter, and always thereafter, give a positive Elsberg reaction when injected with the red corpuscles of his blood. And, finally—

4. If the mere fact of cohabitation, without offspring, between a man and woman would produce Elsberg's reaction in the woman when injected with the red corpuscles of her male participant.

These experiments are reported to have given the following, somewhat astonishing results:

1. Three pregnant women, injected with emulsionized red corpuscles from their husbands, gave a positive Elsberg reaction.

2. One of the above women, injected with erythrocytes from her husband, and at the same time from a stranger, gave a positive reaction with the former and a negative reaction with the latter.

3. Thirteen pregnant women were injected with the emulsion from males with whom they had never cohabited, twelve of whom gave negative results, whilst in one the reaction was doubtful.

4. Corpuscles from two husbands gave positive reaction when injected into their pregnant wives, but negative results in three other pregnant women whom they had never known.

5. In five non-pregnant women who had previously borne children to their husbands, there were negative results in four, and a doubtful reaction in one.

Had Mayoral and Jimenez extended their inquiries to the children of the husbands whose blood induced positive reactions in their pregnant wives, the interest in the matter might have been greatly accentuated, with the possibility that the practical value of their findings would have become correspondingly enhanced, more especially if they had found that their children reacted as did their mothers. As the matter now stands, aside from their scientific worth as aids in determining, at a much earlier date than is now possible, the existence, or not, of pregnancy, and with it, in case of doubt, it may be, the daddy of the child, also, it would be difficult, perhaps impossible, to estimate the true value of these experiments, should they be confirmed by other experimenters—a consummation which is highly improbable, to say the least. Nevertheless, Mayoral and Jimenez are to be congratulated on the originality of their researches and for having given the profession a new cud on which it may chew for some time. Chew it!

R. H. L. Bibb.

